# Global Trend on Machine Learning in *Helicobacter* within One Decade: A Scientometric Study

**DOI:** 10.1155/2023/8856736

**Published:** 2023-08-12

**Authors:** Omid Eslami, Mohsen Nakhaie, Mohammad Rezaei Zadeh Rukerd, Maryam Azimi, Ellahe Shahabi, Amin Honarmand, Mahdiyeh Khazaneha

**Affiliations:** ^1^Gastroenterology and Hepatology Research Center, Institute of Basic and Clinical Physiology Sciences, Kerman University of Medical Sciences, Kerman, Iran; ^2^Clinical Research Development Unit, Afzalipour Hospital, Kerman University of Medical Sciences, Kerman, Iran; ^3^Department of Traditional Medicine, School of Persian Medicine, Kerman University of Medical Sciences, Kerman, Iran; ^4^Faculty of Management and Economics, Shahid Bahonar University, Kerman, Iran; ^5^Department of Emergency Medicine, Afzalipour Hospital, Kerman University of Medical Sciences, Kerman, Iran; ^6^Neurology Research Center, Kerman University of Medical Sciences, Kerman, Iran

## Abstract

**Purpose:**

This study aims to create a science map, provide structural analysis, investigate evolution, and identify new trends in *Helicobacter pylori* (*H. pylori*) research articles.

**Methods:**

All *Helicobacter* publications were gathered from the Web of Science (WoS) database from August 2010 to 2021. The data were required for bibliometric analysis. The bibliometric analysis was performed with Bibliometrix R Tool. Bibliometric data were analyzed using the Bibliometrix Biblioshiny R-package software.

**Results:**

A total of 17,413 articles were reviewed and analyzed, with descriptive characteristics of the *H. pylori* literature included. In journals, 21,102 keywords plus and 20,490 author keywords were reported. These articles were also written by 56,106 different authors, with 262 being single-author articles. Most authors' abstracts, titles, and keywords included “Helicobacter-pylori.” Since 2010, the total number of *H. pylori*-related publications has been decreasing. Gut, PLOS ONE, and Gastroenterology are the most influential *H. pylori* journals, according to source impact. China, the United States, and Japan are the countries with most affiliations and subjects. In addition, Seoul National University has published the most articles about *H. pylori*. According to the cloud word plot, the authors' most frequently used keywords are gastric cancer (GC), *H. pylori*, gastritis, eradication, and inflammation. “*Helicobacter pylori*” and “infection” have the steepest slopes in terms of the upward trend of words used in articles from 2010 to 2021. Subjects such as GC, intestinal metaplasia, epidemiology, peptic ulcer, eradication, and clarithromycin are included in the diagram's motor theme section, according to strategic diagrams. According to the thematic evolution map, topics such as *Helicobacter pylori* infection, B-cell lymphoma, CagA, *Helicobacter pylori*, and infection were largely discussed between 2010 and 2015. From 2016 to 2021, the top topics covered included *Helicobacter pylori*, *H. pylori* infection, and infection.

## 1. Introduction

*Helicobacter pylori* (*H. pylori*) is a spiral gram-negative bacterium located on the epithelial surface of the stomach [1]. The prevalence of *H. pylori* infection ranges from 20% to 50% in developed countries to more than 80% in developing countries [2]. Although the vast majority of infected individuals will never develop *H. pylori*-related symptoms [3], an enormous amount of evidence suggests that *H. pylori* is always a pathogen that all populations would be better off without and that it should therefore be eradicated [4].

*H. pylori* infection risk factors include low socioeconomic status [5], an increase in the number of siblings [6], and having an infected parent, especially an infected mother [7]. This infection is typically contracted in the early years of life and lasts indefinitely unless treated, causing chronic inflammation of the underlying mucosa [8]. Therefore, *H. pylori* infection has been associated with gastric and duodenal ulcers (1–10% of infected patients), gastric cancer (GC) (0.1–3%), and gastric mucosa-associated lymphoid tissue lymphoma (less than 0.01%) [9]. *H. pylori* is a known carcinogen for the development of GC, as the second leading cause of cancer-related deaths worldwide [10]. Furthermore, there is evidence linking *H. pylori* to the etiology of otherwise unexplained iron deficiency anemia, idiopathic thrombocytopenic purpura (ITP), and vitamin B12 deficiency. Under these circumstances, *H. pylori* needs to be identified and eradicated [9–11].

Based on the evidence, it is estimated that the relationship between this microbe and its host dates back 100,000 years [12]. In the past five years, numerous studies have investigated *H. pylori* as an important pathogen. According to published data, the majority of these studies focused on six broad categories: genetics, enzymes, antioxidants, microbial community, nanoparticles, and amino acids. Genetic diversity is one of the most important factors that contribute to the survival and virulence of *H. pylori* [13]. Multiple enzymes, including urease, catalase, lipase, phospholipase, and protease, are used by *H. pylori* to ensure microbial virulence [14]. Several medicinal plants and chemicals have been studied as antioxidants, which inhibit the oxidation reaction and the production of free radicals in the body, thereby preventing the damaging impacts of *H. pylori* [15, 16]. The microbial community as another influential factor during the last 5-year studies indicates that *H. pylori* can induce changes in microbial populations in favor of infection and pathogenicity progression [17]. Nanoparticles, which are small particles ranging in size from 1 to 100 nanometers [18], have been identified as one of the *H. pylori* treatment options [19]. Finally, amino acids, which have been discussed in fewer articles, play a dual role in *H. pylori* infection. Some of amino acids, such as alanine, can contribute to infection progression, while others, such as L-tyrosine, have an inhibitory effect [20].

## 2. Literature Review

Mahala and Singh analyzed research output in Indian universities by retrieving 26,173 documents, including conference proceedings, journal articles, and review papers. It was discovered that science research outputs have been steadily increasing, with the University of Delhi (DU) having the most publications. Furthermore, multiauthored articles were found to have a higher research impact because they were cited more frequently than single-authored papers. The United States, South Korea, and Germany collaborated most with Indian universities, such as Anna University, Indian Institute of Technology, and the Center for Scientific and Industrial Research (CSIR) of India. This study also revealed the recent growth trend of top Indian universities. The findings could be used to help determine where to focus efforts in order to increase scientific research output even more [21].

In a study by Suk et al., 37,451 publications were displayed during the 18-year study period, including 19,080 articles, 10,396 conference abstracts, 2,625 reviews, 1,943 proceedings, and 1,866 letters. In the Science Citation Index, 1,727 journals were listed in 122 subject categories. The fields of clinical gastroenterology, hepatology, microbiology, pharmacology, and pharmacy have conducted the majority of *H. pylori* research, and the G7 developed countries produced the majority of the world's total output. The term “ulcer” remains a hotspot in *H. pylori* research, and the use of “gastric cancer” has increased over the 18-year study period [22].

Suk and et al. presented another study on classic *H. pylori* papers in the Web of Science (WoS). The Science Citation Index Expanded was used in this study, which contains 59,904 documents related to *H. pylori* publications on the Web of Science from 1900 to 2016 to identify classic papers. This article presents classic *H. pylori* papers with at least 1,000 citations from the Web of Science Core Collection published between 1991 and 2007, sorted by publication language, document type, publication output, and journal availability. The output distribution and lifecycle of Web of Science categories, publications by author, institution, country, and citations are used to evaluate classic documents. Among these papers, the study by Parsonet et al. was the most cited paper in the Web of Science Core Collection, from the date of its initial publication until the end of 2016. Finally, the findings revealed that the *H. pylori* study's three main research foci were gastric cancer, low-grade gastric lymphoma, and the CagA gene [23].

In their scientometric study on gastritis in G20 countries, Amanullah and Rajeswari used data obtained from the Web of Science and analyzed them using HistCite and VOSviewer. The results show that Malfertheiner, Graham, and Megraud are their three most cited authors, having been cited 4,933, 4,588, and 4,129 times, respectively. According to journal analysis, the Gut journal (8,320 citations) is the most cited, followed by World Journal of Gastroenterology (6,603), Proceedings of the National Academy of Sciences of the United States of America (4,050), Infection and Immunity, Gastroenterology (3,749), and Journal of Immunology (3,613). In accordance with an institutional citation analysis, Vanderbilt University is the most cited (5952 citations), followed by Baylor College of Medicine (5,061), Magdeburg University (3,953), Hokkaido University (3,264), Aberdeen University (3,212), INSERM (2,722), and Tokyo University (2,594) [24].

Moradi et al. conducted a case study of the D8 countries to assess studies on health science based on the quadruple helix model using content analysis as well as altmetric and scientometric indicators from the Altmetrics.com and WOS databases. The findings revealed that the majority of interactions occurred in the innovation and knowledge spaces, with quadruple imbalances observed in the countries studied. Furthermore, certain interactions occurred across all social networks, demonstrating that users in the D8 countries are concerned with health-related issues. Clinical sciences had the most interaction across all four spaces. In the current approach, this model has never been used with altmetric data [25].

Sahoo and Pandey showed that 53.57% (8,195) of the investigated research documents were published on open-access platforms. In analyzing the researchers' journal preferences, Journal of Virology was identified as the most preferred journal, producing approximately 5.48% (839) of the articles. The research output was found to be dominated by China and the United States, with the University of Hong Kong producing the highest percentage of research papers (3.58%, *n* = 547). The majority of research documents were published in medicine (49.70%), followed by immunology and microbiology (35.72%). Biochemistry, genetics, and molecular biology were the other preferred domains (22.32%). Since January 2020, an unprecedented number of publications have been devoted to COVID-19, and there has been a significant increase in research findings in this area [26].

Mariam et al. carried out a meta-analysis with a scientometric review to investigate the evolution of research on the safety and health of women in the construction industry. They attempted to identify research trends and patterns, as well as hazards and workplace stressors faced by women working in the construction industry. Their findings revealed five major recurring themes: workplace psychological health, HIV/AIDS and construction work, occupational health and safety injuries, gender inclusivity and sexism in construction, and gender-specific health and safety analysis. The main dangers that women faced in the construction industry were biological in nature. Their results also revealed that research on women's health and safety is progressing slowly, with the United States, Japan, Australia, and South Africa leading the way in terms of research development [27].

Today, machine learning assists scientists in numerous fields of study [28]. Also, in medicine, scientific topics can be investigated using co-occurrence analysis, and their relationship can be gleaned directly from the subject content. *R* was created by statisticians for statisticians, but any developer can predict the same trend by looking at its syntax. *R* is the right choice for anyone who wants to gain a better understanding of the underlying details and be innovative because it contains mathematical computations involved in machine learning, which are derived from statistics. *R* is also the best choice for analyzing a corpus of texts by deconstructing paragraphs into words or phrases to identify patterns. *H. pylori* is one of the medical research topics of interest. Because of the importance of *H. pylori* infection in terms of patient quality of life and complications, this study aims to conduct bibliometrics of *H. pylori* infection and its related components in the literature. Therefore, the purpose of this research is to generate a science map, offer a structural analysis, explore evolution, and identify new trends regarding *H. pylori* treatment and prevention in the published research between 2010 and 2021.

## 3. Methods

### 3.1. Data Collection

In 2021, all *H. pylori*-related publications were collected from all WoS databases, such as Medline, Biosis, and Core Collection. The search strategy was as follows: (TS = ((helicobacter OR gastrospirillum) OR (helicobacter OR gastrospirillum))) AND LANGUAGE: (English) AND DOCUMENT TYPES: (article), timespan: 2010–2021.

**Figure 1:**
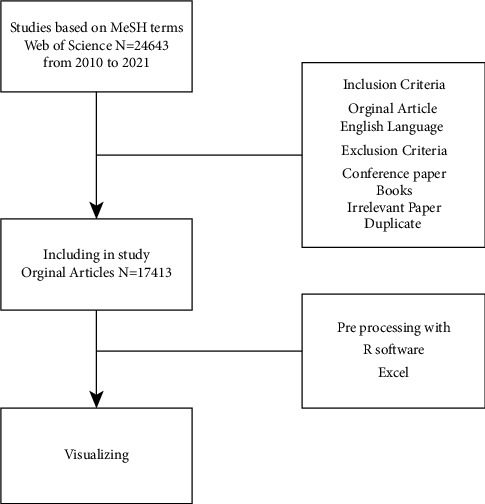
Flowchart of data collection of *Helicobacter pylori*.

*H. pylori* data were collected from the WoS using a search strategy that spanned ten years, from August 2010 to 2021. The information has been obtained using Boolean operators based on keywords, titles, and abstracts. However, nonrelated conference abstracts, books, and articles were excluded. Since the majority of the terms in this field were in English, the search strategy was restricted to articles published in English in all WoS databases. The data required for bibliometric analysis, such as authors, year of publication, journal, affiliation, country of origin, organizations, financing organization, and keywords, were extracted as a plain text file from the database, with no ethical validation required (Figure 1).

### 3.2. Bibliometric Analysis

The bibliometric analysis was performed with Bibliometrix R Tool [29]. Bibliometric data were analyzed using the Bibliometrix Biblioshiny R-package software (https://bibliometrix.org/biblioshiny.html) [30]. The R tool is an open-source tool for scientific map analysis and plotting. It is widely regarded as the most powerful and adaptable open-source statistical software [31]. This bibliometric analysis aimed to visualize, identify, and describe data based on [32]:

1. Annual scientific production and year of publication
2. Sources and documents published on the subject
3. Authors and institutions that work in this field
4. Countries that have played a role in publishing these articles
5. Keywords, subjects, and themes in this field

### 3.3. Data Analysis

Coword analysis was used to analyze keyword co-occurrences as well as identify relationships and interactions between research topics and emerging research trends. Also, the content analysis technique was used to map the strength of association between keywords in textual data [33]. This technique measures the co-occurrence of keywords to examine content in the textual data.

## 4. Results

### 4.1. Main Information

A total of 17,413 articles were reviewed and analyzed (Figure 1), with descriptive characteristics of the articles published in the *H. pylori* literature shown in Table 1. In this descriptive analysis, 21,102 keywords plus and 20,490 authors keywords were reported in journals. These articles were also published by 56,106 different authors, of which 262 were single-author articles. The co-authorship rate was reported to be 3.27, indicating a relatively high level of collaboration. The number of documents per author was reported to be 0.31, with nearly all three authors contributing to an article (Table 1).

### 4.2. Annual Scientific Productions

**Figure 2:**
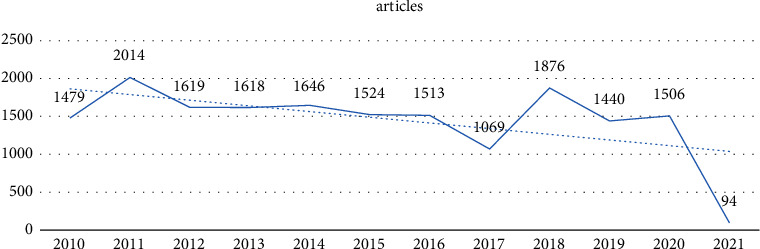
Annual scientific productions.

The annual scientific production on *H. pylori* infection is shown in Figure 2. As can be seen, the number of articles published increased from 2010 to 2011, followed by a decrease from 2011 to 2017. There is a dramatic decline in the number of published articles in 2017, possibly due to the use of more effective drug regimens, higher success rates in treating *H. pylori* infection, and reduced complications. In contrast, the number of articles published in 2018 increased, indicating that researchers focused on topics such as drug resistance as well as basic issues such as genetics, enzymatic discussions, antioxidant activity, microbial connections, and amino acids [34]. Finally, the number of *H. pylori*-related articles has been declining since 2019.

### 4.3. Core Journals on the Influential Aspects of *Helicobacter pylori*

The source impact and the Bradford law were both used to determine the original and influential journals contributing to the *H. pylori* literature.

The ranking of journals is shown in Table 2 based on the h-, m-, and g-indices, total citations (TCs), and the number of publications (NP). Based on these criteria, Gut, PLOS ONE, and Gastroenterology are the most influential journals contributing to the *H. pylori* literature.

Bradford's law of variance is the law of diminishing returns and variance. Bradford wrote that he enacted the act in 1948 and asserted that, for certain subjects, “there are some very prolific journals, many more modest producers, and many more in ever-decreasing production.” The Bradford law divides journals into three zones. For each issue or topic, the top one-third (zone 1 or core) represents the journals that are most frequently cited in the literature on that topic and are therefore most likely to be of interest to researchers in the field. The middle third (zone 2) contains journals with an average citation frequency, whereas the bottom third (zone 3 or tail) contains journals that are rarely cited and of marginal importance to the field. Zone 1 contains the most important journals in this field. There are 43 journals in zone 1, and the remaining journals are located in zones 2 and 3. The top twenty journals in zone 1 are shown in Table 3.

### 4.4. A Three-Field Plot of *Helicobacter pylori* Publications

**Figure 3:**
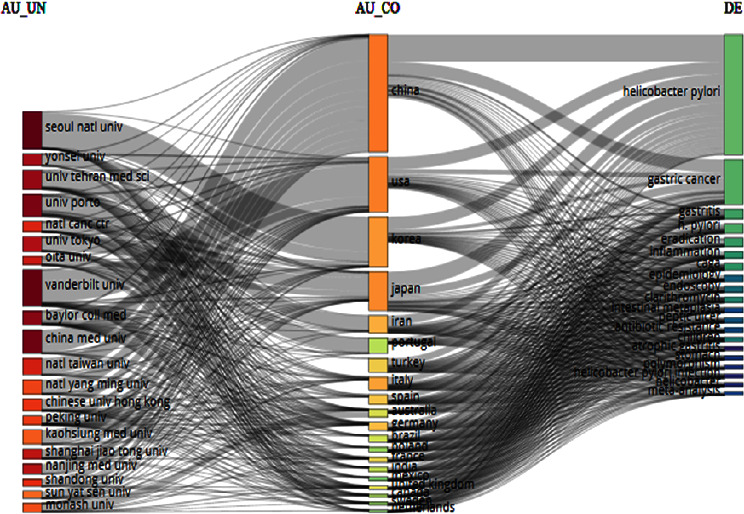
The three-fold analysis of the *H. pylori* publications.

A three-fold analysis of *H. pylori* publications is shown in Figure 3 in terms of affiliation, country, and keyword plus. Rectangles of various colors were used to explain the diagram's relevant elements. The height of the rectangles was determined by the sum of the relations originating between the elements that the rectangle represented (one of the elements: authors, countries, and keywords).

China has most collaborations with most affiliations and subjects, followed by the United States and Japan. *H. pylori* subjects have been considered in most countries and affiliations. Moreover, Seoul National University has published the highest number of articles on *H. pylori*. So, Seoul National University and China are the influential university and country for *H. pylori*, respectively.

### 4.5. Core Articles of *Helicobacter pylori* from 2010 to 2021

Table 4 lists the top *H. pylori* articles by total citations published between 2010 and 2021. The following is a summary of the core *H. pylori* articles:

The epidemiological study conducted by Siegel et al. addressed the impact of eliminating socioeconomic and racial disparities on premature cancer-related deaths, with a focus on death rates, cancer incidence over a 12-year period in the United States, and mortality for any reason was standardized by age. According to the findings, the type of cancer and gender can predict the annual incidence of cancer and cancer-related deaths to some extent. Furthermore, the occurrence of cancer types was predicted by gender, race, and the state in which the person lived [35].

Furthermore, Sharma et al. investigated the primary transcriptome of the major human pathogen *H. pylori*, as well as the *H. pylori* genome sequence, and proposed a new model for mapping and annotating the pathogen's initial transcription [36].

The study by Arnold et al. looked at the global incidence of esophageal cancer by a histological subtype in 2012, as well as the rate and pattern of gastric cancer incidence. According to the findings, men are three times more likely than women to develop GC and noncardiac cancer of the stomach which is about 2.1 times more likely than GC [37].

The study by Jakobsson et al. evaluated the short- and long-term effects of clarithromycin and metronidazole, a common treatment regimen for *H. pylori*, on the internal microbiota in the throat and lower intestine. Significant changes in bacterial diversity were observed one week after antibiotic treatment in all treated individuals at both sites. Furthermore, actinobacteria in the throat and feces were significantly reduced immediately after treatment. Although microbiota diversity eventually improved in pretreatment conditions, the microbiota remained abnormal in some cases for up to four years after treatment. Furthermore, high levels of the macrolide resistance gene erm(B) were found four years after treatment, indicating that antibiotic resistance may persist for longer than previously thought. This highlights the importance of limited use of antibiotics to prevent further treatment failure and the possible spread of antibiotic resistance [38].

Moreover, Plummer et al. conducted a synthetic study in 2012 to investigate the global burden of cancers attributable to infections and the effect of infectious agents on the incidence of various cancers. According to the findings, infectious agents cause about 15% of all cancers each year, with *H. pylori* being the most common microbial agent. As a result, detecting and eliminating some infectious agents will be a highly effective method of lowering cancer incidence [39].

Finally, Graham studied *H. pylori* treatment for increasing antibiotic resistance and highlighted the degree of antibiotic resistance to the three-drug regimen, recommending the use of the most effective enzymes in each region with a success rate of more than 90%, based on the prevalence of regional differences [40].

### 4.6. Most Frequent Words in *Helicobacter pylori* Publications

The most common words in *H. pylori* publications are divided into four categories: keywords plus, titles, abstracts, and author's keywords (Tables 5 and 6). As shown in Table 5, the most common occurrences of keyword plus are Helicobacter-pylori, infection, Helicobacter-pylori infection, and expression and the most frequent occurrences words in the titles are Helicobacter, pylori, gastric, and cancer. Furthermore, according to Table 6, the most commonly used words in authors' keywords are Helicobacter, gastric cancer, pylori, and gastritis and the most frequently identified words in abstracts are pylori, gastric, patients, and infection. According to these findings, the majority of authors used the term Helicobacter-pylori in their abstracts, titles, and keywords. The words with a high co-occurrence in the titles, abstracts, and keywords, on the other hand, demonstrate the distinction between the use of words and the significance of the subjects addressed in these sections. The use of generic words in titles, abstracts, and keywords demonstrates the general importance of these understudied topics.

As shown in Table 5, *H. pylori* infection can lead to some complications. Moreover, the only place where this microbe colonizes is the stomach as indicated by the term gastric. *H. pylori*-induced gastritis is the most common gastrointestinal (GI) infection [41]. The association between *H. pylori* infection and GC is well known [42]. Eradication of *H. pylori* infection in asymptomatic patients is quite effective in preventing gastric cancer [43]. *H. pylori* requires the expression of several genes or inflammation markers in order to be pathogenic. In some patients, risk factors contribute to the symptoms or complications associated with *H. pylori* infection. These risk factors are more prevalent in some patients.

### 4.7. Word Cloud in *Helicobacter pylori* from 2010 to 2021

**Figure 4:**
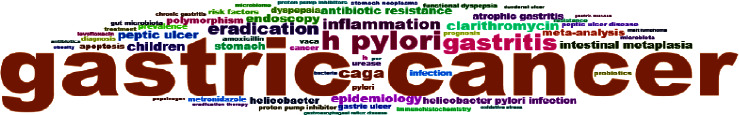
The word cloud of *Helicobacter pylori*.

Figure 4 shows a cloud word plot based on the most frequently used keywords by the authors. Gastric cancer, *H. pylori*, gastritis, eradication, and inflammation are the most frequently used terms.

GC is the fifth most common cancer in the world [44] and the second leading cause of cancer-related death [45]. GC is caused by long-term inflammation of the stomach [46]. *H. pylori* infection is the most important risk factor for GC. People who are infected with *H. pylori* have a much higher incidence of GC than those who are not [47]. *H. pylori* infection leads to many changes in the gastric mucosa, which can lead to complications such as inflammation, immune dysfunction and apoptosis, mitochondrial changes, aging, and gastric dysbacteriosis [48]. *H. pylori* eradication improves the majority of GI symptoms, such as indigestion, abdominal pain, bloating, and decreasing the risk of bleeding in patients with a history of GI bleeding [49, 50], thereby enhancing the quality of life [50].

### 4.8. Word Growth of *Helicobacter pylori* from 2010 to 2021

**Figure 5:**
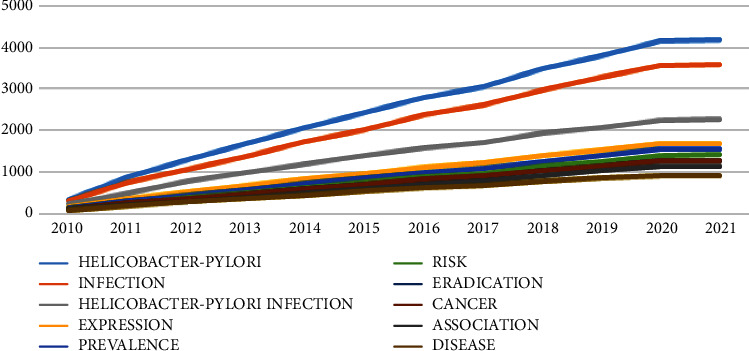
The word growth over time.

Figure 5 illustrates the upward trend of the words used in the articles from 2010 to 2021. As seen in the diagram, the terms *Helicobacter pylori* and infection have the steepest slopes, while the other terms have a less steep slope.

### 4.9. Thematic Map of *Helicobacter pylori* from 2010 to 2021

**Figure 6:**
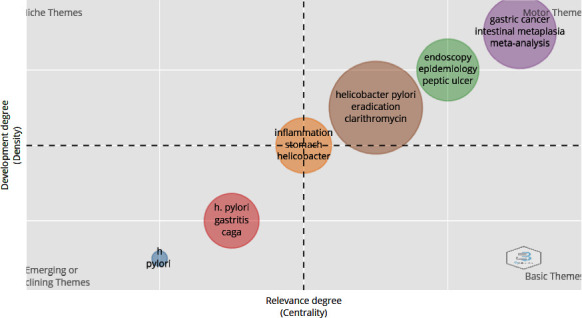
The strategic diagram of *Helicobacter pylori* disease.

Strategic diagrams can be used to assess the significance and progression of subjects.  Figure 6 depicts a strategic diagram of *H. pylori* disease, in which the *x*-axis represents density and the *y*-axis represents the thematic center and divides the diagram into four parts.

The subjects in the bottom left quarters are emerging and declining subjects; new subjects may appear or disappear in this section. The subjects depicted in the lower right quarter are the main traverse subjects, with low density and high centrality, indicating that they have been studied extensively. Furthermore, the subjects in the upper left quarter are denser and less centrality. These topics are well developed but distinct. The subjects shown in the upper right quarter have high density and high centrality. These subjects are developed and essential. This map shows the subjects over a ten-year period. As can be seen, the theme engine and the emerging section have produced the majority of the subjects.

The emerging section of the diagram contains subjects such as *H. pylori*, gastritis, and CagA. In addition, inflammation, stomach, and helicobacter are all found in the center. Furthermore, subjects such as gastric cancer, intestinal metaplasia, epidemiology, peptic ulcer, eradication, and clarithromycin are included in the diagram's motor theme section. In recent years, clarithromycin triple or drug therapy has been one of the recommended treatment regimens for *H. pylori* infection [51]. *H. pylori* infection is the most important risk factor for intestinal metaplasia, a precancerous stage of GC. Eradication of *H. pylori* can improve intestinal metaplasia and thus prevent GC [52]. Since the recent COVID-19 outbreak, a large number of trials have been stopped at an early stage due to limited patient access and the impossibility of feasibility studies, rather than for statistical reasons. As a result, there has been a growing trend in recent years to conduct misanalysis reviews of previous studies' results in the literature [53]. Despite a decrease in the prevalence of *H. pylori* infection and a decrease in duodenal ulcers in recent years, epidemiological studies show that the number of gastric ulcers and GC continues to rise [54].

Numerous studies have found an association between *H. pylori* infection and obesity [55], but some studies have not found an association between *H. pylori* infection and childhood obesity or overweight [56].

*H. pylori* infection can be diagnosed using a number of different approaches. We can classify these techniques as either invasive or noninvasive. In cases that *H. pylori* infection has been treated or the rate of virus colonization is low, immunohistochemistry is a highly sensitive method for diagnosing the infection [57]. To achieve homeostasis in the gastric epithelium, production and death of epithelial cells must be in equilibrium. Numerous studies have demonstrated that infection with *H. pylori* causes limited cell death (apoptosis) [58] or a greater increase in cell proliferation compared to cell death, and this is one of the pathophysiologies that increase the risk of GC [59].

In addition, due to the increasing resistance of antibiotics to treat *H. pylori* infection, day-by-day interest in new treatment regimens with greater efficacy and fewer side effects has grown. One of these techniques is the incorporation of probiotics into treatment regimens, which increases treatment efficacy and decreases treatment-related side effects [60]. Also, all of terms are explained in nodes of Figure 6 which are shown in Table 7.

### 4.10. Thematic Evolution Map of *Helicobacter pylori* Publications

The thematic evolution map shows the historical trend and progression of *H. pylori* infection. Biblioshiny was used to perform thematic evolution, which is displayed in two-time sections. The first section covers the years 2010 to 2015, during which time the articles were analyzed and topics such as *Helicobacter pylori* infection, B-cell lymphoma, CagA, *Helicobacter pylori*, and infection were discussed. *Helicobacter pylori*, *H. pylori* infection, and infection are among the topics covered from 2016 to 2021. In addition, some cases of B-cell lymphoma and CagA have evolved into *Helicobacter pylori* and *H. pylori* infection between 2010 and 2021.

Cancers associated with *H. pylori* have a high incidence compared to other types of cancer. In recent years, however, the evolution of bacterial diagnosis and treatment has advanced due to the development of new techniques [61]. The rarest complication of *H. pylori* infection is B-cell lymphoma. Multiple studies have demonstrated that *H. pylori* eradication can result in regression of primary gastric lymphoma [40]. Due to recent improvements in the diagnostic methods of gastric lymphoma by endoscopy and endosonography and effective treatment regimens for *H. pylori*, the number of articles on lymphoma has decreased.

**Figure 7:**
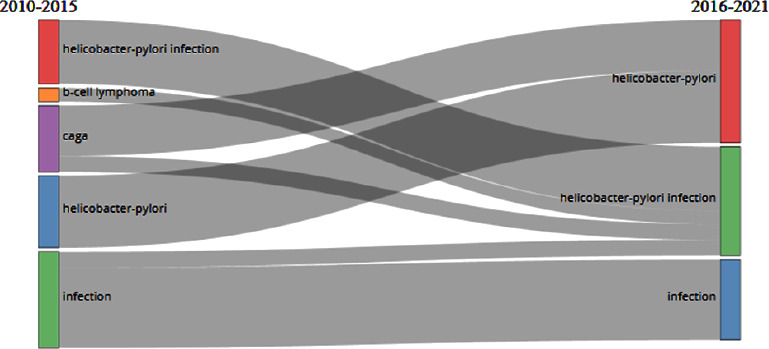
The thematic evaluation map.

Numerous studies have demonstrated that infection with the CagA strain of *H. pylori* increases the risk of complications such as peptic ulcer disease and GC [62, 63]. Given the definitive recognition of the pathogenicity of CagA species and the fact that *H. pylori* treatment is unrelated to the bacterial species, studies on this species have declined in recent years. In addition, each term is defined in the nodes of Figure 7 which are shown in Table 8.

## 5. Discussion

This study analyzed the authors, countries, affiliation, keywords, key research streams, and themes addressed in *Helicobacter*-related articles published in the WoS database between 2010 and 2021 using bibliometric and visualization techniques. During this time, 56,106 authors published 17,413 articles on *Helicobacter* in 2,365 scientific journals. The co-authorship rate was reported to be 3.27, indicating a relatively high level of collaboration. The documents per author were reported to be 0.31, with almost all three authors involved in writing an article. Gut, PLOS ONE, and Gastroenterology are the most important journals contributing to the *H. pylori* literature. This study's data illustrated the progression of studies in recent years and the varying concentrations of evaluations in different years.

*H. pylori* infection is very common, possibly affecting more than half of the world's population [64]. Person-to-person transmission is the most common mode of *H. pylori* infection transmission. Other routes of transmission, such as the consumption of contaminated water or food, may also be involved in the disease's transmission [65]. This infection and its symptoms can reduce the quality of life in patients [50]. Besides, its complications may lead to mortality and shortening of life expectancy in patients. The bacterium's changing epidemiology has been associated with a drop in peptic ulcer disease and GC [66].

There are several methods for diagnosing *H. pylori* infection, which are classified as invasive or noninvasive tests. Endoscopy is used to test tissue samples in invasive methods, but it is not required in noninvasive methods. Immunohistochemistry is a highly sensitive method for diagnosing *H. pylori* infection in treated cases or cases with low bacterial colonization [57].

*H. pylori* is one of the most enduring and potent human pathogens. Several strategies have been used to increase its adaptation and survival in the host body [67]. One of the most important of these strategies relates to high genetic diversity, which exhibits 2.7 to 8% polymorphisms in DNA sequences and its adaptation alongside the evolution of human hosts in various parts of the world, including the United States, Latin America, Europe, East Asia, coastal China, Hong Kong, Japan, South Asia (India), and Africa [67, 68]. On the other hand, increased whole genome sequencing allows for the identification of new strains with greater infectivity as well as a more comprehensive understanding of the structure and function of different parts of the microbe [67]. As a result of the diversity of microbial strains and the breadth of genetics, several studies have addressed microbial genetics in order to better understand microbe behavior and prevent infection.

This systematic review concentrated on studies published in the last ten years. The findings revealed that *H. pylori* is one of the subjects that researchers have always considered, emphasizing the significance of *H. pylori* infection. However, a review of annual scientific production revealed that the number of studies addressing *H. pylori* has decreased sharply since 2019 and the start of the global COVID 19 pandemic, owing to medical researchers' focus on COVID-19 disease, its related complications, ways to treat and control COVID-19 disease, and the impact of this pandemic on most medical issues, including *H. pylori*.

Gut, PLOS ONE, and Gastroenterology have published the most articles about *H. pylori*. Furthermore, China, the United States, and Japan published most *H. pylori*-related articles. Some of the most cited articles have been those focusing on epidemiological topics and GC. *H. pylori* infection, a bacterial carcinogen, is the greatest risk factor for GC, a disease that claims hundreds of thousands of lives annually [69]. Due to this, the majority of articles on GC have addressed *H. pylori* infection, and cancer has been mentioned more frequently than other terms (after the keywords Helicobacter, infection, and gastritis) in *H. pylori*-related articles.

The relationship between *H. pylori* infection and gastric malt lymphoma (GML) is well established, and the incidence of GML has decreased in recent years due to earlier diagnosis. According to the findings of this study, the number of meta-analysis studies has increased in recent years as a result of the COVID-19 pandemic, decreased outpatient visits, travel restrictions, home quarantines, delays in elective care, and decreased patient access. This study also revealed that most articles focused on new treatment regimens with lower resistance and greater efficacy in eradicating *H. pylori*, such as the use of probiotics. Furthermore, the number of articles focusing on subjects such as B-cell lymphoma and CagA has decreased in recent years due to a better understanding of the pathophysiology of *H. pylori* infection, more effective treatments, and the role of species such as CagA in bacterial pathogenesis.

The role of enzymes in establishing *H. pylori* infection through colonization, damage to the host epithelium, and provision of essential metabolic substrates as one of the most important virulence factors has shifted researchers' attention to the association of microbes with various enzymes in recent publications [70]. Urease, for instance, is one of the most important enzymes produced by *H. pylori* in order to protect the microbe from the acidic conditions of the stomach and its mucous membranes at low pH [69, 71]. Catalase is an additional enzyme produced by this microorganism that contributes to its growth and survival at inflammatory surfaces by catalyzing the removal of toxic oxygen species [72].

As antioxidants can prevent the production of DNA-damaging free radicals and the treatment of H. pylori infection is one of the most important topics related to this microbe, numerous studies have examined antioxidants as a means to prevent chronic infection with *H. pylori* and reduce the risk of GC and its treatment [15, 16]. Ngan et al. reported, for instance, that *Hericium erinaceus* possesses antioxidant properties that can prevent *H. pylori* infection [73].

In *H. pylori* infection, the microbial community can cause changes by influencing the population of gastric microbes [17]. By altering the microbiota of the gastric mucosa, this microorganism may influence T-reg cell synthesis and secretion. It also attempts to maintain itself and infection by inhibiting immune responses and gastritis function [74]. Due to the complex relationship between *H. pylori* and other microorganisms and the increased risk of GC, several studies have attempted to identify the microbial population affected by *H. pylori* in the context of infectivity and tumorigenesis; however, additional research is necessary to investigate this topic [75].

Both nanoparticles and amino acids have received less research. The issue of nanoparticles is associated with therapeutic topics. In addition, bismuth with two antibiotics metronidazole and tetracycline or metronidazole and amoxicillin is the most effective treatment to prevent *H. pylori* infection. On the other hand, one of the primary disadvantages of nanoparticles is their lack of shared characteristics; consequently, nanoparticles have likely been the subject of fewer studies [76, 77]. Since amino acids are the only source of carbon energy for *H. pylori*, numerous studies have demonstrated that the majority of amino acids promote microbial activity and infectivity, while only a few, such as L-tyrosine, inhibit bacterial activity [20, 78, 79]. Therefore, because amino acids do not have a complex relationship with *H. pylori*, they are not considered interesting topics for the study by researchers.

## 6. Conclusion

This study identified significant and influential aspects of the *Helicobacter pylori* literature published between 2010 and 2021. These influential characteristics will have an effect on future core investigations. In this study, researchers examined 17,413 published articles by 56,106 authors. This study revealed that Gut, PLOS ONE, and Gastroenterology are the most influential journals for *H. pylori* research. Journals comply with Bradford's law, and 43 journals are classified in zone 1 and are more important than others. In addition, Seoul National University has the most published papers on *H. pylori*. Lastly, Seoul National University and China are the influential *Helicobacter* university and nation, respectively.

*H. pylori* infection can lead to several complications. Furthermore, the only place this organism colonizes is the stomach, as indicated by the term gastric. *H. pylori* requires expression of several genes or inflammatory markers that establish the virulence of this bacterium. Risk factors cause symptoms or complications associated with *H. pylori*.

Due to its complications, *H. pylori* infection remains an important and challenging issue in the healthcare systems of all nations and costs a significant amount of money annually. It is well known that pathogenic strains such as CagA, VacA, and HtrA, activation of pathways such as NF-kappa B, and induction of tumor necrosis factor alpha protein contribute to the pathogenicity of *H. pylori* infection.

Future research into the targeted treatment of pathogenic strains or the treatment and control of pathogenic pathways and proteins may be of great assistance in reducing the disease's complications. In the last two years, as a result of the global COVID-19 epidemic, the number of studies on *H. pylori* has decreased significantly, and the consequences may result in an increase in complications such as GI bleeding and GC in the coming years. Thus, researchers in various fields of clinical and basic sciences should shift their attention to various facets of this significant bacterium.

This scientometric review offers useful reference points for early-stage researchers and provides valuable in-depth information to experienced researchers and practitioners in the field of *H. pylori* research. This scientometric analysis and visualization are promising for comprehensively reflecting the global picture of classification-based *H. pylori* research and have potential for visualizing the emerging trends in other research fields [80].

Nonetheless, our research had some limitations. Our study's main limitation was a lack of access to the full text of a large number of articles. This cause, combined with a lack of funds, posed numerous challenges for our team in moving the project forward. An important point to consider as a future direction in upcoming projects is the relationship between *H. pylori* and the type of treatment described in the literature due to multiple antibiotic resistance.

## Figures and Tables

**Table 1:** The descriptive characteristics of the *H. pylori* publications.

Description	Results
Timespan	2010 : 2021
Sources (journals)	2,365
Documents	17,413
Average years from publication	6.12
Average citations per document	20.73
Average citations per year per doc	2.805
References	307,278
Article	17,138
Keywords plus (ID)	21,102
Author's keywords (DE)	20,490
Authors	56,106
Author appearances	125,481
Authors of single-authored documents	262
Authors of multiauthored documents	55,844
Single-authored documents	324
Documents per author	0.31
Authors per document	3.22
Co-authors per documents	7.21
Collaboration index	3.27

**Table 2:** The top journals based on the source impact.

Journals	h-index	g-index	m-index	TC	NP	PY-start
Gut	57	100	4.75	10,338	122	2010
PLOS ONE	54	78	4.5	14,623	630	2010
Gastroenterology	53	87	4.416667	7,856	106	2010
World Journal of Gastroenterology	49	70	4.083333	11,994	513	2010
Helicobacter	46	64	3.833333	12,468	716	2010
Infection and Immunity	38	51	3.166667	4,037	137	2010
Proceedings of the National Academy of Sciences of the United States of America	38	68	3.166667	4,701	75	2010
Alimentary Pharmacology & Therapeutics	36	51	3	3,823	124	2010
International Journal of Cancer	36	53	3	3,477	107	2010
American Journal of Gastroenterology	34	50	2.833333	2,683	62	2010

h-index: the number of articles from that journal that has at least the same number of cited and referenced articles, g-index: gives more weight to highly cited journals, m-index: takes into account years since the first publication and is more relevant to an earlier career journal than the h-index, TC: times cited, NP: number of publications, and PY-start: year start.

**Table 3:** Journal rankings according to the Bradford law.

Journals	Rank	Citation freq	cumFreq	Zone
Helicobacter	1	744	744	Zone 1
PLOS ONE	2	645	1389	Zone 1
World Journal of Gastroenterology	3	520	1909	Zone 1
Scientific Reports	4	251	2160	Zone 1
Journal of Gastroenterology and Hepatology	5	216	2376	Zone 1
Digestive Diseases and Sciences	6	191	2567	Zone 1
Infection and Immunity	7	138	2705	Zone 1
Alimentary Pharmacology & Therapeutics	8	124	2829	Zone 1
BMC Gastroenterology	9	123	2952	Zone 1
Gut	10	122	3074	Zone 1
Frontiers in Microbiology	11	119	3193	Zone 1
Gastroenterology Research and Practice	12	111	3304	Zone 1
Journal of Bacteriology	13	110	3414	Zone 1
International Journal of Cancer	14	109	3523	Zone 1
Journal of Gastroenterology	15	109	3632	Zone 1
Gastroenterology	16	107	3739	Zone 1
Scandinavian Journal of Gastroenterology	17	105	3844	Zone 1
Medicine	18	104	3948	Zone 1
Gut and Liver	19	97	4045	Zone 1
Asian Pacific Journal of Cancer Prevention	20	94	4139	Zone 1

Citation freq: citation frequency; cumFreq: cumulative frequency.

**Table 4:** Most globally cited papers per year.

Papers	Total citations	TC per year
Siegel RL, 2011, CA Cancer J clin	28,081	2.55*E* + 03
Siegel RL, 2017, CA Cancer J clin	2,406	4.81*E* + 02
De Martel C, 2012, lancet oncol	1,238	1.24*E* + 02
Tacconelli E, 2018, lancet infect dis	1,186	2.96*E* + 02
Kostic AD, 2012, genome res	955	9.55*E* + 01
Sharma CM, 2010, nature	791	6.59*E* + 01
Arnold M, 2015, gut	651	9.30*E* + 01
Siegel RL, 2020, CA Cancer J clin	634	3.17*E* + 02
Mechanick JI, 2013, obesity	630	7.00*E* + 01
Jakobsson HE, 2010, plos one	605	5.04*E* + 01
Plummer M, 2016, lancet glob health	596	9.93*E* + 01
Graham DY, 2010, gut	592	4.93*E* + 01
Megraud F, 2013, gut	566	6.29*E* + 01
Sugano K, 2015, gut	556	7.94*E* + 01
Song F, 2010, health technol asses	519	4.32*E* + 01
Mannoor MS, 2012, nat commun	514	5.14*E* + 01
Thun MJ, 2010, carcinogenesis	509	4.24*E* + 01
Supuran CT, 2010, bioorg med chem lett	492	4.10*E* + 01
Stanghellini V, 2016, gastroenterology	471	7.85*E* + 01

TC: total citation.

**Table 5:** The most frequently used words in keyword plus and titles.

Words	Occurrences
*Keyword plus*	
Helicobacter-pylori	4,199
Infection	3,587
Helicobacter-pylori infection	2,265
Expression	1,681
Prevalence	1,562
Risk	1,413
Eradication	1,277
Cancer	1,271
Association	1,148
Disease	912
Gastric-cancer	832
Identification	780
Management	771
Inflammation	766
Meta-analysis	766
Epidemiology	758
Carcinoma	755
Escherichia-coli	736
Population	680
Protein	677

*Title*	
Helicobacter	7,929
Pylori	7,332
Gastric	4,753
Cancer	2,467
Infection	2,331
Patients	1,710
Study	1,430
Risk	1,153
Eradication	1,009
Cells	931
Therapy	922
Expression	755
Disease	746
Association	692
Gastritis	672
Human	633
Analysis	604
Treatment	580
Cell	576

**Table 6:** The most frequently used words used in the author's keywords and abstracts.

Words	Occurrences
*Authors' keyword*	
Helicobacter-pylori	5,245
Gastric cancer	1,648
Pylori	529
Gastritis	446
Eradication	317
CagA	306
Inflammation	293
Clarithromycin	252
Children	232
Endoscopy	223
Epidemiology	222
Peptic ulcer	219
Antibiotic resistance	206
Intestinal metaplasia	204
Meta-analysis	193
Stomach	190
Polymorphism	180
Helicobacter	179
Helicobacter pylori infection	177
Atrophic gastritis	172

*Abstract*	
Pylori	43,231
Gastric	29,727
Patients	26,944
Infection	17,392
Cancer	15,470
Study	14,285
Helicobacter	14,130
Results	12,002
Cells	10,119
Risk	9,185
Expression	8,414
Eradication	8,247
Significantly	7,332
Treatment	7,191
Therapy	6,918
Methods	6,635
Ci	6,465
Analysis	6,456
Gastritis	6,069
Cell	5,864

**Table 7:** The thematic table of related words.

Words	Cluster label
Duodenal ulcer, obesity, functional dyspepsia, proton pump inhibitor, peptic ulcer disease, risk factor, prevalence, dyspepsia, Helicobacter-pylori infection	Endoscopy
Peptic ulcer, epidemiology, endoscopy, pepsinogen, chronic gastritis, immunohistochemistry, stomach neoplasms, prognosis, apoptosis, atrophic gastritis, polymorphism, meta-analysis, intestinal metaplasia	Endoscopy
CagA, gastritis, H. pylori, PCR, VacA	*H. pylori*
Antibiotics, eradication, therapy, levofloxacin, proton pump inhibitors, resistance, treatment, amoxicillin, metronidazole, urease, clarithromycin, children, antibiotic resistance	*Helicobacter pylori*
Bacteria, microbiome, microbiota, gut microbiota, probiotics, cancer, infection, helicobacter, stomach, inflammation	Inflammation

**Table 8:** The thematic evaluation of *H. pylori*.

From	To	Words	Weighted inclusion index	Inclusion index	Occurrences	Stability index
B-cell lymphoma, 2010–2015	*Helicobacter pylori* infection, 2016–2021	B-cell lymphoma	0.15	0.25	104	0.01
CagA, 2010–2015	*Helicobacter pylori*, 2016–2021	CagA; VacA	0.57	0.25	274	0.01
CagA, 2010–2015	*Helicobacter pylori* infection, 2016–2021	Genotypes; clinical-relevance	0.18	0.25	78	0.01
*Helicobacter pylori*, 2010–2015	*Helicobacter pylori*, 2016–2021	Helicobacter-pylori; expression; Escherichia-coli; identification; inflammation; protein; gene; cells; activation; in-vitro; epithelial-cells; colonization; gene-expression; pathogenesis; NF-kappa-b; growth; mice; inhibition; gastric epithelial-cells; apoptosis; diversity; crystal-structure; inflammatory-bowel-disease; acid; induction; binding; sequence; genes; system; mechanisms; oxidative stress; proteins; receptor; necrosis-factor-alpha; survival; DNA; mechanism; immune-response; virulence factors; virulence; secretion; iv secretion; T-cells; bacteria; vacuolating; cytotoxin; pathogenicity island; ulcerative-colitis; in-vivo; responses; urease; crohns-disease; pylori; helicobacter; evolution; gastric mucosa; phosphorylation; proliferation; pathway; mouse model; campylobacter-jejuni; metabolism; immune-responses; nitric-oxide; differentiation; dendritic cells; stress; regulatory T-cells; adhesion; model; E-cadherin; mycobacterium-tuberculosis; recognition; pseudomonas-aeruginosa; immunity; motility; Staphylococcus-aureus; rats; campylobacter; interleukin-8; translocation; recombination; derivatives; invasion	0.82	0.01	2428	0.00
*Helicobacter pylori*, 2010–2015	*Helicobacter pylori* infection, 2016–2021	Mucosa; pylori infection	0.02	0.01	236	0.01
*Helicobacter pylori* infection, 2010–2015	*Helicobacter pylori*, 2016–2021	Cancer; carcinogenesis; colorectal-cancer; breast-cancer; progression; hepatocellular-carcinoma; aberrant DNA methylation; DNA methylation	0.09	0.01	697	0.01
*Helicobacter pylori* infection, 2010–2015	*Helicobacter pylori* infection, 2016–2021	Helicobacter-pylori infection; risk; association; disease; carcinoma; gastric cancer; population; epidemiology; risk factors; stomach; intestinal metaplasia; peptic ulcer; adenocarcinoma; atrophic gastritis; classification; gastroesophageal-reflux disease; helicobacter-pylori eradication; nonsteroidal anti-inflammatory drugs; follow-up; united-states; antibodies; symptoms; prevention; stomach-cancer; mortality; polymorphisms; increased risk; diseases; lesions; body-mass index; barretts-esophagus; seroprevalence; cohort; irritable-bowel-syndrome; seropositivity; smoking; age; squamous-cell carcinoma; endoscopy; esophageal; obesity; atrophy; features; health; impact; trends	0.72	0.01	1398	0.01
*Helicobacter pylori* infection, 2010–2015	Infection, 2016–2021	Randomized controlled-trial; Japan	0.01	0.02	94	0.01
Infection, 2010–2015	*Helicobacter pylori* infection, 2016–2021	Prevalence; diagnosis; children; gastritis; duodenal ulcer; peptic-ulcer disease; transmission; adults; functional dyspepsia	0.18	0.02	860	0.01
InfecHion, 2010–2015	Infection, 2016–2021	Infection; eradication; management; meta-analysis; strains; therapy; antibiotic resistance; triple therapy; clarithromycin; resistance; susceptibility; metronidazole; omeprazole; amoxicillin; double-blind; efficacy; ulcer; mutations; eradication therapy; antimicrobial resistance; proton pump inhibitor; quadruple therapy; proton pump inhibitors; PCR; polymerase-chain-reaction; trial; consensus report; sequential therapy; clarithromycin resistance; guidelines; multicenter; lansoprazole; levofloxacin; c-13-urea breath test; antimicrobial susceptibility	0.91	0.02	2027	0.01

## Data Availability

Data are available from the corresponding author upon reasonable request.
